# Patients’ physiological reactions to competitive rehabilitation therapies assisted by robotic devices

**DOI:** 10.1186/s12984-023-01163-2

**Published:** 2023-04-11

**Authors:** José M. Catalán, Andrea Blanco-Ivorra, José V. García-Pérez, Yolanda Vales, David Martínez-Pascual, Santiago Ezquerro, Alicia Garrote, Teresa Costa, Luis D. Lledó, Nicolás García-Aracil

**Affiliations:** 1 Robotics and Artificial Intelligence Group of the Bioengineering Institute, Miguel Hernández University, Avda. de la Universidad, 03202 Elche, Spain; 2 Hospital la Pedrera, Dénia, Spain

**Keywords:** Rehabilitation, Multiplayer games, Interpersonal rehabilitation games, Stroke, Patient engagement, Exergames, Robotics

## Abstract

**Background:**

The aging of the population and the progressive increase in life expectancy in developed countries is leading to a high incidence of cerebrovascular diseases. Several studies have demonstrated that robot-assisted rehabilitation therapies combined with serious games can improve rehabilitation outcomes. Social interaction in the form of multiplayer games has been highlighted as a potential element to increase patient’s motivation and exercise intensity, which professionals have described as one of the determining factors in maximizing rehabilitation outcomes. Despite this, it has not been widely studied. Physiological measures have been proven as an objective tool to evaluate patients’ experience in robot-assisted rehabilitation environments. However, they have not been used to evaluate patients’ experience in multiplayer robot-assisted rehabilitation therapies. The main objective of this study is to analyze whether the interpersonal interaction inherent in a competitive game mode affects the patients’ physiological responses in robot-assisted rehabilitation environments.

**Methods:**

A total of 14 patients participated in this study. The results of a competitive game mode were compared with a single-player game mode with different difficulty levels. Exercise intensity and performance were measured through parameters extracted from the game and the information provided by the robotic rehabilitation platforms. The physiological response of patients in each game mode was measured by the heart rate (HR) and the galvanic skin response (GSR). Patients were asked to fill out the IMI and the overall experience questionnaire.

**Results:**

The exercise intensity results show that high-difficulty single-player game mode is similar in terms of intensity level to a competitive game mode, based on velocity values, reaction time and questionnaire results. However, the results of the physiological responses of the patients measured by GSR and HR are lower in the case of the competitive mode compared to the high-difficulty single-player game mode, obtaining results similar to those obtained in the low-difficulty single-player game mode.

**Conclusions:**

Patients find the competitive game mode the most fun, which is also the mode they report experiencing the most effort and stress level. However, this subjective evaluation is not in line with the results of physiological responses. This study concludes that interpersonal interaction inherent to a competitive game mode influences patients’ physiological responses. This could mean that social interaction is an important factor to consider when interpreting the results obtained from physiological measurements.

## Background

According to World Population Prospects 2022 of the United Nations, the proportion of persons aged 65 or over is projected to increase globally between 2022 and 2050. At the world level in 2022, approximately 10 percent of people are aged 65 or over [1]. The proportion of older persons in the world is projected to reach nearly 12 percent in 2030, and 16 percent in 2050 [1]. This segment of the population is particularly prone to suffer acquired brain injury (ABI) like a cerebrovascular accident (CVA) or stroke. The relative incidence of stroke doubles every decade after age 55, and even higher when coming to the current COVID-19 pandemic and potential long-term neurologic effects, called as long COVID [2]. Improvements in treatments and early detection have increased the survival rate, so the number of people who have survived a CVA has increased. In most cases, ABI or stroke causes a severe disruption in the patient’s daily life, as it can lead to physical, cognitive, emotional, and social deficits. Impairments of dexterous upper-limb function are a significant cause of disability following an ABI or stroke since they affect approximately one-half of the patients in this clinical population [3]. A proper recovery program and an appropriate intensity level can help hemiparetic patients regain movement in their affected limbs [4–6].

The needs presented by these people make the care and rehabilitation exercises provided by robotic rehabilitation platforms even more important today and in the coming years [7]. Several studies have demonstrated that robot-assisted rehabilitation therapies combined with serious games can improve rehabilitation outcomes [8–10]. However, it is unclear which aspects of robot-assisted rehabilitation therapies must be improved to maximize rehabilitation outcomes for stroke patients [10]. The general consensus on stroke rehabilitation is that virtual therapy assisted by robotic devices is a good way to increase motivation and exercise intensity, which professionals have described as determining factors in the outcome of rehabilitation [11, 12]. Furthermore, increasing patient motivation has been shown to improve patients’ adherence to rehabilitation treatment [12].

Difficulty adaptation algorithms are one of the most promising elements for maximizing motivation and intensity over the long term [13–16]. There have also been efforts to improve motivation through audiovisual elements, scores, and/or by proposing cognitive challenges [15, 17, 18]. However, an under-utilized resource in stroke rehabilitation assisted by robotic devices is social interaction as a factor that increases patients’ motivation and, therefore, the intensity of therapy. Social interaction in the form of multiplayer games has been highlighted as a potential element to increase patient motivation in motor rehabilitation therapies, both a therapist playing with a patient [19–21], and two patients [22–26]. Most studies evaluating multiplayer therapies present preliminary results in the current state of the art. In addition, they do not typically involve two robotic rehabilitation platforms to assist patients in evaluating multiplayer therapies. Most studies employ rehabilitation technologies that cannot assist patients when they cannot perform the exercise movements by themselves. Therefore, the inclusion criteria of the studies avoid recruiting patients who need assistance to perform the exercises.

Generally, the game experience can be measured by physiological measures and/or questionnaires. There are numerous examples in the literature of the use of physiological measures to assess the gaming experience of patients in single-player rehabilitation therapies. The overall conclusion is that they significantly improve the evaluation of the gaming experience [18, 27]. Physiological measures have been successfully used to estimate the cognitive and affective state of patients during therapy, which has been used not only to assess the game experience but also to develop difficulty adaptation algorithms that take into account the affective state of the patient [28–32].

In the context of difficulty adaptation algorithms based on physiological measures for competitive or cooperative games, some methods have been evaluated in recent years [33–36]. However, developing such a difficult adaptation algorithm for multiplayer games is a complex problem that still needs to be effectively solved. In addition, it is not common to use physiological measures to evaluate the game experience in multiplayer rehabilitation games [34]. The lack of studies using the information provided by physiological signals in multiplayer rehabilitation games makes assessing the game experience difficult.

We have previously conducted a study with unimpaired participants to evaluate how the interpersonal interaction inherent in a competitive game mode affects the physiological response of participants [37]. Ten unimpaired participants were involved in this study (5 pairs). Different therapy sessions were defined: i) a free session without a competitor, ii) two sessions with a virtual competitor with different difficulty levels, iii) a competitive game. The physiological measures showed differences between the competitive mode and a high-difficulty single-player game mode mainly due to the interpersonal interaction inherent in the competitive game mode. These differences might impact how the information provided by physiological signals is interpreted in this type of therapy.

The main objective of this study is to research whether the interpersonal interaction inherent in a competitive game affects the physiological response of patients in robot-assisted neurorehabilitation therapy. For this purpose, the results of a competitive game mode were compared with a single-player game mode with different difficulty levels. The aim is to define a single-player game mode similar to the competitive mode in all aspects so that the differences observed are not due to a different level of demand but to the interpersonal interaction involved in competing against a real competitor.

## Methods

### Participants

14 patients participated in this study, whose demographics, impairment type and clinical scales are listed in Table 1. Before entering the study, all patients provided written informed consent. Inclusion criteria were: (i) adults with hemiparesis after a non-traumatic brain injury; (ii) those with the cognitive capacity to understand study instructions and perform the task; (iii) those who benefit from the technology; (iv) inpatients at the Pedreda hospital. Exclusion criteria were: (i) people with hemiplegia and spasticity (modified ashworth scale (MAS)>1) in the upper limb; (ii) people with painful shoulder; (iii) people with severe perceptual linguistics deficits (Wernicke’s aphasia); (iv) people with visual deficits (apperceptive visual agnosia); (v) those who withheld consent or were unable to give consent (verbal or written). All 14 patients completed the entire study.

The study was developed in La Pedrera Hospital (Denia, Spain). The study protocol complied with the Ethical Committee of La Pedrera Hospital. Hospital therapists selected participants. They were matched according to similar conditions, based on the degree of independence in activities of daily living (ADL), the upper limb muscle condition, and the level of cognitive impairment. To assess these aspects, the hospital therapists have relied on the information provided by the clinical scales. However, their experience working with these patients in the hospital has also been considered when making the pairings. The degree of independence in ADL was evaluated through the Barthel Index as follows [38, 39]: 100 points, independent (90 points if the patient uses a wheelchair); 60 points, mild dependencies; 35–55 points, moderate dependency; 20–35 points, severe dependency; and a score of less than 20 points, total dependency. Muscular conditions and functional mobility of the upper limb were evaluated through a specific test for neurological injuries such as the stroke rehabilitation assessment of movement (STREAM) [40]. The maximum score for this test is 16 points. The STREAM is used to assess the active range of motion and quality of movement. The information provided by STREAM was used to support the configuration of the different difficulty levels of the game and the assistance levels. The short portable mental status questionnaire (SPMSQ) is a screening test used to detect cognitive impairment [41]. The scoring of this questionnaire is interpreted as follows: 0–2, normal cognitive functioning; 3–4, mild cognitive impairment; 5–7, moderate cognitive impairment; and 8, severe cognitive impairment. The information provided by SPMSQ was used to determine whether patients’ cognitive capabilities were sufficient for the study.

**Table 1:** Characteristics of the patients

n	Pair ID	Sex	Age	Diagnostic	Laterality	Barthel Index	STREAM	SPMSQ
1	P1	F	77	Ischemic stroke in ACM	Right	70/90	11/16	2/8
2		M	63	Ischemic stroke in ACM	Right	70/90	8/16	3/8
3	P2	F	69	Ischemic stroke in ACM	Right	40/90	2/16	NA
4		M	71	Ischemic stroke in ACM	Right	40/90	1/16	5/8
5	P3	M	65	Ischemic stroke in ACM	Right	1/90	2/16	2/8
6		F	84	Bihemispheric ischemic stroke	bilateral	25/90	7/16	0/8
7	P4	M	62	ICH stroke in thalamus mesencephalus	Left	95/90	16/16	0/8
8		M	69	Basal ganglia stroke	Right	20/90	13/16	1/8
9	P5	F	56	Frontotemporal haemorrhage	Left	10/90	13/16	6/8
10		M	61	Carotid ischemic stroke	Left	25/90	N/A	N/A
11	P6	F	78	Basal ganglia haemorrhage	Right	20/90	0/16	2/8
12		M	63	Guillain Barré syndrome	N/A	0/90	9/16	N/A
13	P7	M	78	Ischemic stroke in ACM	Right	60/90	13/16	6/8
14		M	68	Thalamic lacunar stroke	Left	65/90	N/A	4/8

*Pair ID* identifier of the pair of patients who competed in the competitive mode (CM), *ACM* Artery cerebral middle,*Barthel Index* is an index for assessing independence in activities of daily living [38, 39]; *ICH*,Intracerebral hemorrhage; *N/A*, not available value; *STREAM* Stroke rehabilitation assessment of movement, the value shown indicates the score obtained for the upper limb movement [40]; *SPMSQ*, Short portable mental status questionnaire is a screening questionnaire used to detect cognitive impairment [41]; *PJI* Periprosthetic joint infection

### Rehabilitation robots

Two Rubidium devices were used in this study [19, 42, 43]. Rubidium is commercialized and distributed by the spin-off iDRhA [44]. This robotic rehabilitation platform is a portable, desktop-type, upper-limb rehabilitation robotic device with two actuated degrees of freedom. It consists of an articulated-parallelogram mechanism driven by two electric motors that perform movements in a horizontal plane.

**Fig. 1:**
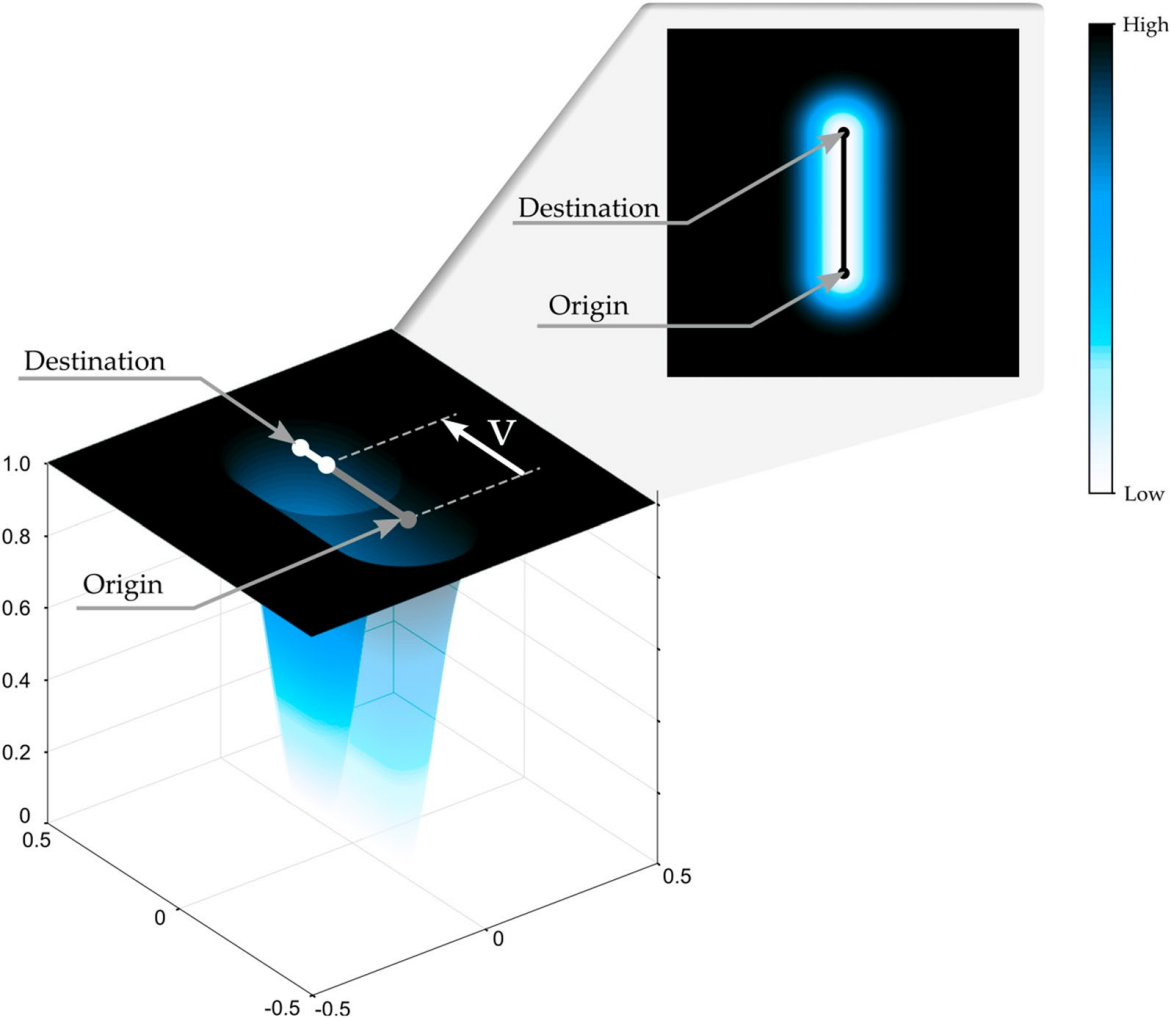
Graphic representation of the force fields used in this study. The scale represents the normalized assistance force magnitude applied in each position. The maximum assistance force value is configured by means of the assistance level of the robotic rehabilitation device

In this study, we used a force-field-based assistance mode of the Rubidium robotic rehabilitation platform. Figure 1 shows the version of the force field used in this study. This tunnel-shaped force field with ends helps the patient perform the movement linearly while assisting the patient in reaching the target if necessary. The tunnel’s origin point corresponds to the patient’s initial position, while the destination point corresponds to the target to be reached. After a delay, the origin point of the tunnel starts to move towards the target at a constant velocity that depends on the maximum time configured in the game to reach the target successfully. In this study, a delay of 1 s has been configured. This method assists only when the patients cannot move toward the target at a velocity greater than the minimum velocity required, allowing them to move freely toward the target in case no assistance is needed. This force-field-based assistance mode has already been validated in other studies with patients [19, 45].

### Experimental setup

**Fig. 2:**
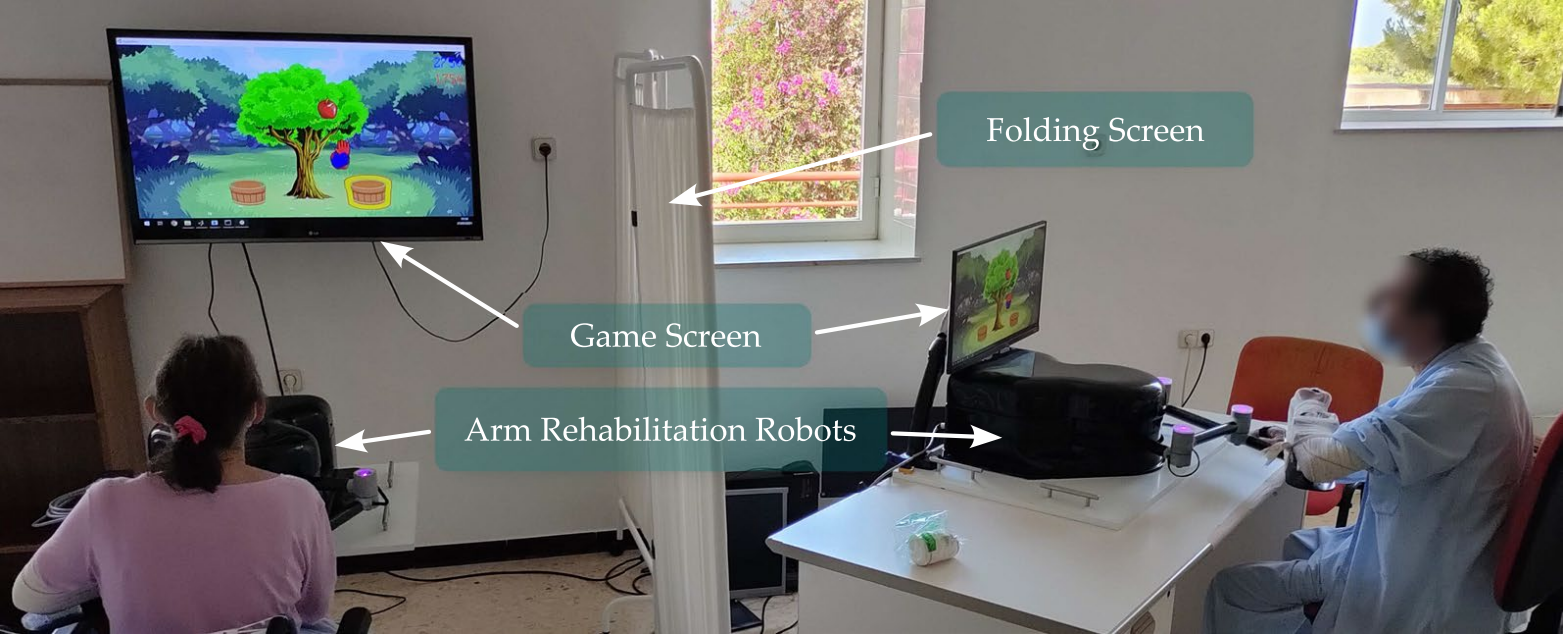
Overview of the experimental setup

Both patients were seated in front of a robotic rehabilitation device (Fig. 2). The patient rests the arm on an orthosis anchored to the end effector, supporting the weight of the forearm. In front of each of them, there was a screen on which the game was shown. Participants were instructed by their therapists not to interact each other during the sessions. For this reason, a panel was placed to prevent them from seeing each other during the sessions in order to limit their interaction.

People in wheelchairs who cannot sit at a table may have problems using the Rubidium rehabilitation platform because the wheelchair may collide with the table, so they cannot get close enough to use the device properly. For this reason, a Rubidium device with a lifting structure was used to facilitate its use with patients sitting in wheelchairs. The other Rubidium was placed directly on a table. For this reason, both screens used have different sizes. The screen for the device placed on a table is smaller because it is closer to the patient. While the Rubidium device adapted for wheelchair use has a larger screen because it is hung on the wall farther away from the patient. Before carrying out the study, we checked that the size and distance of the screens were adequate for the patients to perform the exercises correctly.

### Game

**Fig. 3:**
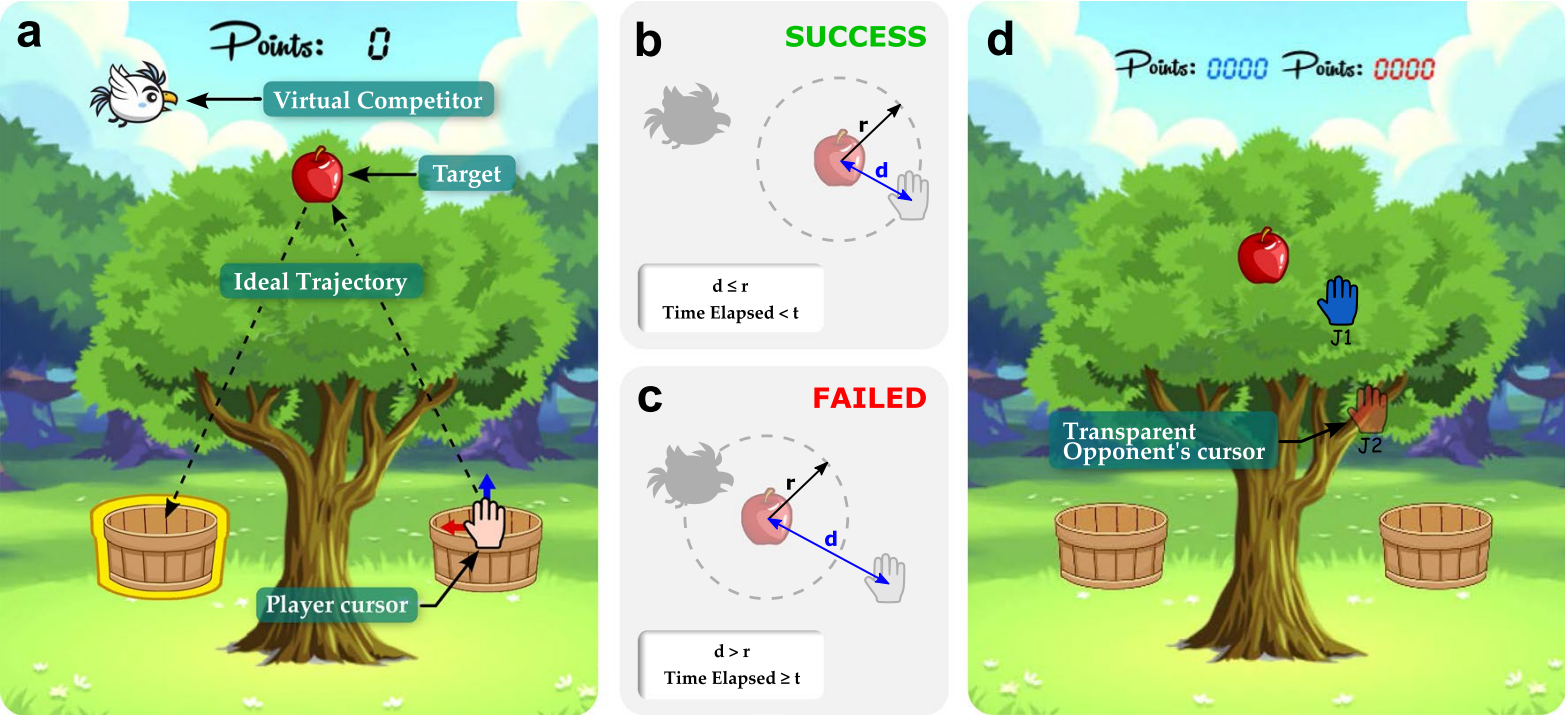
Overview of the game. **a** Single-player game mode. **b** Condition to reach the target successfully, where *d* is the distance to the target, *r* is the minimum distance to reach the target successfully, and t is the maximum time to reach the target. **c** Condition to fail to reach the target. **d** Competitive game mode

The game used in this study is the same as the one analyzed in [37], whose purpose is to reach a target (an apple) and deposit it in the indicated position (highlighted basket). It is, therefore a point-to-point modality where the player’s cursor (patient) was represented by a hand whose center corresponds to the actual position of the robot end-effector. The patient was considered to reach a target when the distance *d* from his/her cursor to the apple was less than or equal to the distance *r* (Fig. 3b). In this study, the distance *r* was set to 1 cm in all cases. The time *t* (Fig. 3b and Fig. 3c) was the amount of time the user had to reach the target before the bird reached it.

As in [37], two game modalities were carried out:

- On the one hand, a single-player game mode, where a bird represented a virtual competitor. First, the player had to wait in the basket until an apple appeared (Fig. 3a) and then try to reach it faster than the bird to drop it in the indicated basket. The bird always appears when an apple appears. However, the time to wait in the basket until an apple appears is pseudo-random so that patients are alert and avoid anticipation. The movement speed of the bird is constant and depends on the time set to reach the apples successfully. Within the single-player game mode, there were two different game modalities: low-difficulty mode and high-difficulty mode. The difficulty level in each case was adjusted by the *t* parameter.
- On the other hand, a competitive game mode, in which two players participated simultaneously (Fig. 3d). Patients played against each other in pairs and tried to score more points than the other. Points were assigned on a first-come, first-served basis, so patients had to pick up the apple and put it in the basket before their competitor. During the game, patients could see the other player’s scores and position throughout the game.

### Outcome measures

#### Estimation of the exercise intensity

The velocity of the hand has been proven to be a reasonable estimation of energy consumption during arm rehabilitation therapies in post-stroke patients [23–25]. In particular, estimation of the energy expenditure based on the root mean square (RMS) velocity value of the hand is correlated with other estimations methods such as those base on heart rate response, electromyography activity, or oxygen consumption [46, 47]. Consequently, this measure can be used as an objective measure related to exercise intensity. Rubidium rehabilitation platform provides real-time end-effector velocity value. It has been computed RMS value of the hand velocity from the speed profiles defined by the patients in every trailer. Mean and maximum hand velocity values have also been extracted to have more information to discuss the differences between conditions in exercise intensity.

Another parameter related to exercise intensity is the reaction time. As mentioned above, the difficulty level is adjusted by increasing/decreasing the time to reach the target and the assistance level. In this type of exercise, speed is correlated with reaction time. This is because if you have less time to reach the targets, you must move faster and react sooner. Therefore, reaction time correlates with exercise intensity. Reaction time was measured as the time that elapses from when the apple appears until the patient begins to move.

#### Estimation of the exercise performance

In this study, the game score (success rate) was used to estimate the exercise performance of the patients in every condition. The game score is closely related to the difficulty level of the exercise. It allows us to evaluate whether or not the patients are able to perform the exercise correctly with a certain difficulty level. For this reason, the game score has been widely used to estimate the exercise performance in virtual reality game-based neurorehabilitation therapies [13, 24, 25, 33, 48].

#### Measurement of the physiological response

One of the measured physiological responses was the galvanic skin response (GSR). is a standard measure in psychophysiological paradigms, and therefore it is often used in affective state detection [27]. For this purpose, two Shimmer3 GSR+ units from Shimmer were used, one for each participant. These devices have a built-in signal-processing unit responsible for taking the measurement, cleaning the signal, and transmitting it via Bluetooth. The sample rate of this sensor unit is 50 Hz. From the GSR signal provided by these sensors, skin conductance response (SCR) was computed. Two reusable electrodes placed on the proximal phalanges of the index and middle fingers of the hand not used to control the robot (non-dominant hand) were used to measure the GSR signal.

Another standard measurement in rehabilitation games is the heart rate (HR) [27]. Two Zephyr BioHarness$^{\hbox {TM}}$ (Zephyr Technology Corporation) were used for this purpose. This device has an internal unit responsible for measuring, processing, and transmitting via Bluetooth the electrocardiogram (ECG) of the user in real-time. The sampling rate of this device is 250 Hz. HR was extracted from the ECG.

To analyze how the game mode affected the user’s affective state, the values reached at the end of each game mode for each physiological feature were evaluated.

#### Subjective assessment of the experience

The overall experience questionnaire was used to assess the preferred game mode of the patients. The version used in this study is similar to the versions used in other studies [13, 24, 25, 49]. This questionnaire asks patients to compare playing alone and playing with someone else by means of three questions:

1. Which game mode did you prefer? (5 options: strongly/weakly preferred playing alone, no preference, weakly/strongly preferred playing with someone else)
2. Which game mode was more fun? (7 options: playing alone was slightly/moderately/much more fun, both were equally fun, playing with someone else was slightly/moderately/much more fun).
3. In which game mode did you feel more tension? (7 options: playing alone was slightly/moderately/much more tense, both were equally tense, playing with someone else was slightly/moderately/much more tense)

Several assessment tools are currently available to evaluate patient motivation and satisfaction during technology-assisted rehabilitation. One of the most commonly used tools is the intrinsic motivation inventory (IMI) [50, 51]. This subjective questionnaire measures four aspects of engagement: enjoyment/interest, effort/importance, perceived competence, and pressure/tension. There are several versions of this questionnaire [52]. The one used in this study is a reduced version that has already been evaluated in similar studies [22–24, 37].

### Study protocol

**Fig. 4:**
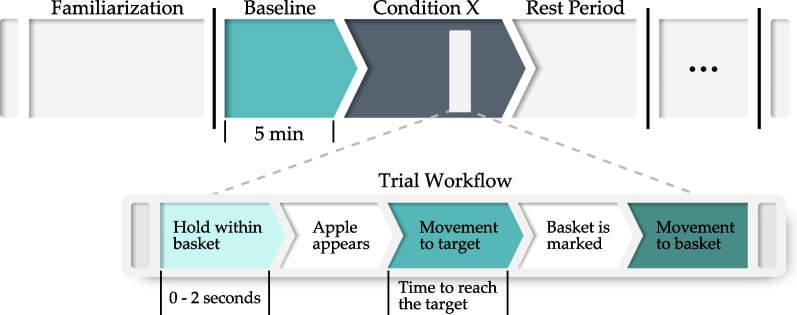
Diagram summarizing the study protocol

Figure 4 shows a diagram summarizing the protocol followed in the study. Upon arrival, what the study consisted of was explained to each patient. After that, they were asked to move in front of the robot and equipped with the physiological sensors. patients are familiarized with the system and receive precise instructions about the actions to perform the exercise correctly.

While providing instructions to the user, an evaluation of the patients’ skills is carried out to define the different difficulty levels of the single-player game modes based on the assistance level and the maximum time to reach the targets. During the evaluation period, the hospital therapists collaborated to define an appropriate difficulty level based on these two parameters. The difficulty level set in low-difficulty mode (LDM) was challenging but low enough to reach the targets easily. This difficulty level corresponds to that usually configured by therapists in a conventional robot-assisted therapy with the Rubidium rehabilitation platform. In the case of high-difficulty mode (HDM), a more challenging difficulty level was defined, where it would be difficult to reach the targets but not so difficult as to demotivate the patient.

After that, experimentation begins. The procedure is the same for all game modes (Fig. 4). First of all, a period of 5 min was dedicated to obtaining the patients’ baseline for each physiological signal. Then, both patients perform the exercise simultaneously. The order in which each pair of patients performs the conditions are established randomly in advance. Finally, there is a rest period. During this rest period, patients filled out the IMI questionnaire, where they rated, according to their subjective experience, each of the game modalities according to their preference. This process is repeated 3 times, once for each game mode. At the end of the study, patients fill out the overall experience questionnaire.

In this study, all game modalities were configured with 35 objectives, in other words, 35 trials. The duration of the session is not predetermined. It depends on the speed of the patients in reaching the targets. Each of the apples appears at a random time between 0 to 2 s. The movement range of the game has been set to 20 cm (10 cm radius).

Participants could not see each other during the sessions and were instructed not to interact. However, they were allowed to interact between conditions. By limiting patient interaction, we aim to reduce the variability in the results due to, for example, different interpersonal relationships. Furthermore, we want to assess whether this factor still influences the results despite reducing all external interactions, with only the interpersonal interaction inherent in a competitive game mode.

### Data analysis

A normality test was carried out using the Shapiro-Wilk test. Results show evidence that parameters are not normally distributed. Friedman test was employed to study differences between conditions. Therefore, differences between groups were studied using the Friedman test. In the post-hoc analysis, pairwise comparisons were studied by the Wilcoxon signed-rank test with the zero method proposed by Pratt [53]. In addition, the Holm-Bonferroni method was used to control the family-wise error rate.

The velocity values were normalized by the minimum forward speed (forward speed of the tunnel-shaped force-field-based assistance mode) (Eq. 1, where $x_{min}$ corresponds with the forward speed of the tunnel-shaped force-field-based assistance mode) The results obtained from the physiological signals (GSR and HR) were normalized to the baseline value measured just before each session (Eq. 1, where $x_{min}$ corresponds with the baseline value). This normalization method has already been used in other similar studies [37, 54, 55]. Statistical analysis was performed using normalized values.

Results of the assistance level were normalized to the one provided in LDM (Eq. 1, where $x_{min}$ corresponds with the assistance level provided in LDM). The assistance level provided at LDM corresponds to the assistance level therapists of La Pedrera Hospital, usually configured in a conventional robot-assisted therapy with the Rubidium rehabilitation platform.

1 $$\begin{aligned} x_{norm} = \frac{x - x_{min}}{x_{min}} \end{aligned}$$

## Results

### Exercise intensity

**Fig. 5:**
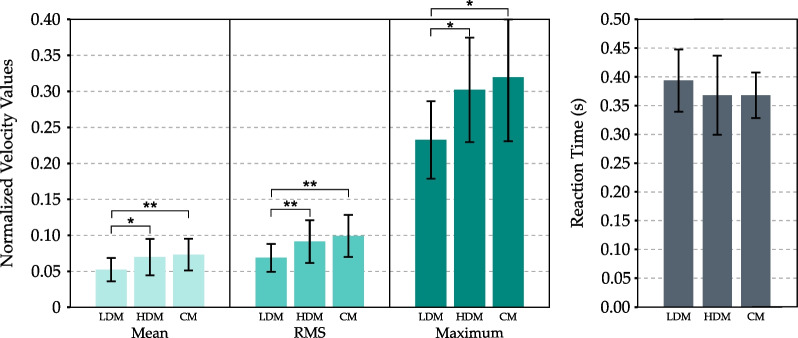
Exercise intensity parameters. The velocity values were normalized so that a value equal to 0 corresponds to the minimum forward speed of the tunnel-shaped force-field-based assistance mode (Eq. 1). Bars represent mean values, while error bars represent standard deviations. Statistical differences are represented by * ( $p<=0.05$), ** ($p<=0.01$) *** ($p<=0.001$) and **** ($p<=0.0001$)

Parameters directly related to the exercise intensity are shown in Fig. 5. The RMS velocity value shows significant differences between conditions (Friedman Test $p = 0.0015$). Pairwise comparisons show that velocity values in the HDM are significantly higher than in the LDM ($p = 0.0086$). In addition, results indicate that RMS velocity values in CM are significantly higher than LDM ($p = 0.0037$), but it is practically the same as the one obtained in the HDM.

In terms of mean velocity value, results also show significant differences between conditions (Friedman Test $p = 0.0003$). In this case, it is also observed that values in the HDM and the CM are significantly higher than those obtained in LDM ($p = 0.015$ and $p = 0.003$ respectively), but they are similar to each other. Statistical study of the maximum velocity values also shows similar results.

On the other hand, reaction time results show no significant differences between conditions (Friedman Test $p = 0.42$).

### Assistance Level

**Fig. 6:**
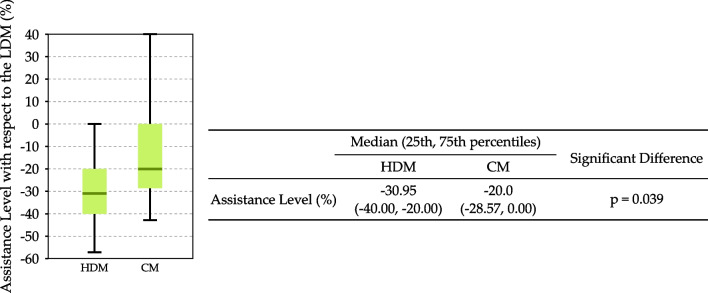
Assistance level provided to patients relative to that provided in low-difficulty mode (LDM). The table collects the median value with the first and third quartiles and the p-value of the statistical study performed. The value provided corresponds to the percentage of variation of the assistance force provided in Newtons

Figure 6 graphically represents the assistance level provided to patients relative to the one provided in LDM. The assistance level in the HDM is about 30.95% lower than in LDM, significantly lower than that provided in the competitive mode, which was about 20% lower than in LDM.

### Exercise performance

**Fig. 7:**
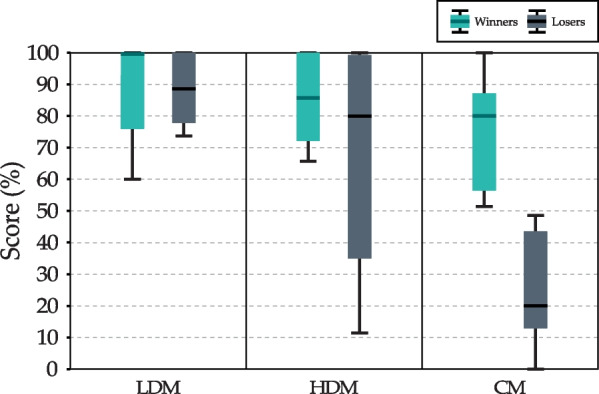
Representation of the game score, divided between winners and losers of the competitive mode (CM). The score is represented as the success rate

The results of the patients’ scores in each game mode are shown in Fig. 7. Results suggest that in single-player games (LDM and HDM) score value decreases when increasing difficulty level. On the other hand, scores differ considerably between winners and losers in CM. Winners reach an average of 48.57% of the targets faster.

### Physiological reaction

**Fig. 8:**
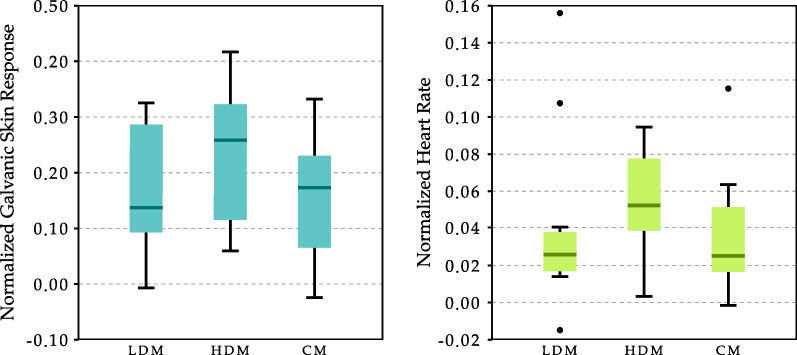
Graphical representation of the results of the galvanic skin response (GSR) and the heart rate (HR). Results have been normalized to the baseline value (Eq. 1)

Results of the measured physiological signals are shown in Fig. 8. In the statistical study results, both GSR and HR show no significant differences between conditions. However, results suggest that in single-player games, the response in both signals tends to increase with the difficulty level, while in the CM, the results obtained are similar to those obtained in the LDM.

Table 2 shows the overall Intrinsic Motivation Inventory results. The results indicate that the interest/enjoyment reported by patients in the CM is higher than in the other conditions. On the other hand, the perceived effort/importance is lower in LDM. Regarding Competence and pressure/tension, no significant differences are observed between conditions. However, results suggest that the highest perceived competence level is reported in CM, while the highest level of pressure/tension is also perceived in the CM.

**Table 2:** Overall Intrinsic Motivation Inventory results. All significant differences between conditions are shown, while all non-significant differences are omitted

	Median (25th, 75th percentiles)			Significant differences
	LDM	HDM	CM	
Interest/enjoyment	0.88	0.88	1.0	
	(0.69, 1.00)	(0.67, 1.00)	(0.83, 1.00)	
Effort/importance	0.83	0.92	0.92	LDM - HDM ($p = 0.027$)
	(0.75 , 0.92)	(0.77, 0.92)	(0.92, 1.00)	
Competence	0.75	0.79	0.92	
	(0.60, 0.98)	(0.58, 1.00)	(0.83, 1.00)	
Pressure/Tension	0.25	0.17	0.33	
	(0, 0.42)	(0, 0.40)	(0.17, 0.42)	

*LDM* low-difficulty mode. *HDM* high-difficulty mode. *CM* competitive mode

### Overall experience questionnaire

**Fig. 9:**
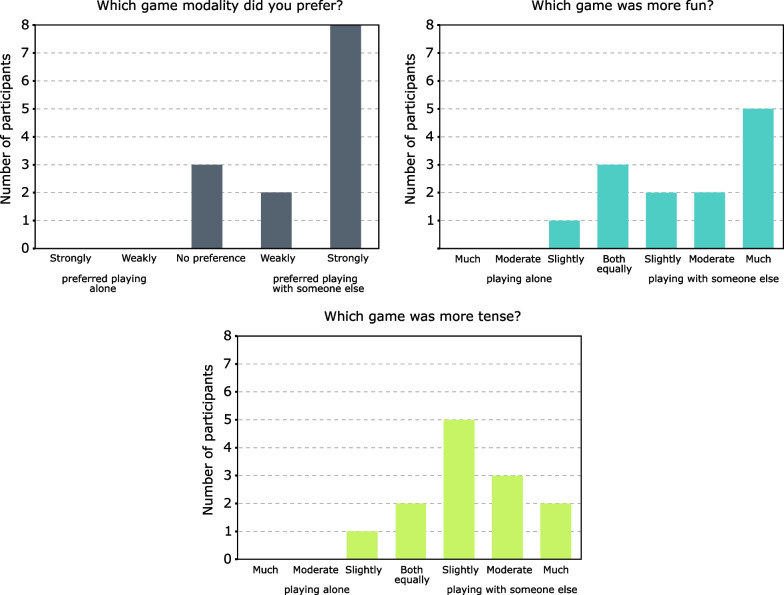
Responses to the overall game experience questionnaire presented with a bar chart per question, indicating the number of subjects who responded to each answer

Results of the overall game experience questionnaire are shown in Fig. 9. Ten patients preferred playing with someone else and 3 had no preference. On the other hand, 9 patients found it more fun to play with their partner, 3 of them enjoyed both equally, and only 1 said he slightly preferred to play alone. Most patients (10 patients) also agree that they have experienced more tension playing with someone else, 2 of them indicate that they have experienced tension both equally, and only 1 says he has experienced slightly more tension playing alone.

## Discussion

### Differences in the exercise intensity

First, we will study the intensity level of each of the game modes. As it can be observed in Fig. 5, the intensity level is higher in the case of the HDM and the CM. On the other hand, although no significant differences are observed in the case of reaction time, it can be observed that the reaction time in LDM is higher than in HDM mode and CM. However, the results in HDM and CM are similar. These results suggest that intensity levels in HDM and CM are similar. At this point, the assistance level provided was analyzed.

Figure 6 shows the assistance level provided by the robotic device in HDM and CM normalized to LDM. The results showed in Fig. 6 suggest that a significantly higher assistance level was provided to patients in CM compared to HDM. Based on these results, we can conclude that patients achieved a reaction time in the CM similar to that in HDM with a higher assistance level.

### Evaluation of the task performance

When we evaluate exercise performance (Fig. 7), we observe that there is a difference between the performance of winners and losers in the CM. As mentioned above, the pairs were formed to couple patients with similar capabilities. In addition, the assistance level was adjusted for each patient to compensate for differences in motor skill levels. However, it is difficult to match patients who can compete on equal terms. Despite these considerations, this is the main reason why there is an appreciable difference between winners and losers. Even so, it can be seen that the winners, in general, do not achieve 100% of the objectives. Therefore, although there is an appreciable difference, a certain level of competitiveness was achieved.

The results show that the patients could reach almost all the targets in the LDM. These results confirm that the difficulty level was properly defined since it allowed patients to reach the targets easily while maintaining a certain difficulty level. In the HDM, patients had a lower score than in the LDM. Those who lost in the CM achieved lower scores in the HDM than the winners. This reflects the difference in the skill level between the winners and losers of the CM.

The IMI results (Table 2) indicate that patients find the CM the most fun game mode (Interest/enjoyment; 1.0 (0.83, 1.00)), which is also the mode they report experiencing the most effort (Effort/importance; 0.92 (0.92, 1.00)) and stress level (Pressure/Tension; 0.33 (0.17, 0.42)). Results indicate not only that they prefer a challenging game mode, but also that patients who lost in CM were not demotivated. In fact, it is in CM that all participants report feeling the most skilled (Competence, 0.92 (0.83, 1.00)). This is another indicator that the pairs were made correctly since a good level of competitiveness was achieved.

The Overall Experience Questionnaire shows similar results. Most participants prefer to play with another person because it is more fun. However, CM is also the game mode in which they felt more stressed.

The results obtained in the questionnaires suggest that the difference we observed in performance is not large enough to cause demotivation in those patients who lost in CM. This indicates that a relatively good level of competitiveness has been maintained.

### Differences in the physiological response

The results demonstrate that LDM and HDM are two significantly different game modalities. However, no significant differences between conditions are observed in the results of physiological reactions. Even so, the results of the physiological reactions for both GSR and HR show a tendency to increase with increasing intensity level.

This was an expected outcome. According to the questionnaire results, patients report that the CM is the mode in which they experience the most effort and stress level (Tab. 2). However, this subjective evaluation is not in line with the results of physiological responses, where they suggest that the physiological response in the CM is somehow lower than in the HDM.

It was shown that HDM and CM are similar in terms of intensity level. Therefore, the difference observed in the physiological responses is not because of the effort required by the exercise since it is similar in both cases. Therefore, the only difference between both conditions is the type of competitor. In the HDM, the patients competed against a virtual competitor (the bird). In contrast, in the CM, although both patients could not communicate with each other outside the game, they were aware that they were competing against a person. Therefore, the difference in physiological responses must be mainly due to this interpersonal interaction caused by playing with a person. We obtained the same result in a previous study conducted with able-bodied participants [37]. We cannot say that this reduction is a positive or negative outcome, but we can consider that there is a difference that could be relevant when discussing the results.

## Conclusions

The results presented in this study prove that high difficulty single-player game mode is similar in terms of difficulty level and intensity to a competitive game mode. Despite this, it was observed that there are differences in the physiological reactions of the patients in the results obtained by the HR and the GSR. It is interesting to note that according to the questionnaire results, patients find the CM the most fun game mode, which is also the mode they report experiencing the most effort and stress level. However, this subjective evaluation is not in line with the results of physiological responses, where the results suggest that the physiological response in the CM is somehow lower than in the HDM. Based on the results obtained in this study, we can conclude that this difference in the patients’ physiological reactions is due to the interpersonal interaction inherent to the competitive game mode. This could mean that social interaction is an important factor to consider when interpreting the results obtained from physiological measurements.

## Data Availability

The datasets generated during and/or analyzed during the current study are available from the corresponding author on reasonable request.

## Abbreviations

ACM: Artery cerebral middle
ABI: Acquired brain injury
ADL: Activities of daily living
CM: Competitive mode
CVA: Cerebrovascular accident
ECG: Electrocardiogram
GSR: Galvanic skin response
HDM: High-difficulty mode
HR: Heart rate
HRQoL: Health-related quality of life
ICH: Intracerebral hemorrhage
IMI: Intrinsic motivation inventory
LDM: Low-difficulty mode
MAS: Modified ashworth scale
PJI: Periprosthetic joint infection
RMS: Root mean square
SCR: Skin conductance response
SPMSQ: Short portable mental status questionnaire
STREAM: Stroke rehabilitation assessment of movement
TBI: Traumatic brain injury
